# Solitary Leptomeningeal Recurrence From Prostate Adenocarcinoma After Treatment With Cytotoxic Gene Therapy, Radiation Therapy, and Androgen Deprivation Therapy

**DOI:** 10.1016/j.adro.2021.100711

**Published:** 2021-05-09

**Authors:** Neil Chevli, Amarinthia E. Curtis, Suzanne Powell, Pamela New, E. Brian Butler, Bin S. Teh

**Affiliations:** a Department of Radiation Oncology, University of Texas Medical Branch at Galveston, Galveston, Texas; b Spartanburg Radiation Oncology, Gibbs Cancer Center, Spartanburg Regional Hospital, Spartanburg, South Carolina; c Departments of Pathology & Genomic Medicine, Houston Methodist Hospital, Houston, Texas; d Department of Neurosurgery, Houston Methodist Hospital, Houston, Texas; e Department of Radiation Oncology, Houston Methodist Hospital, Houston, Texas

## Abstract

**Purpose:**

Leptomeningeal disease in prostate adenocarcinoma is very rare. Solitary leptomeningeal recurrence from prostate adenocarcinoma has only been previously reported once in the published literature.

**Methods and Materials:**

A 63-year-old man with high-risk prostate cancer was treated in a phase I-II trial with androgen deprivation, radiation therapy, and cytotoxic gene therapy. He initially had biochemical control but experienced solitary leptomeningeal recurrence 47 months after diagnosis.

**Results:**

He received androgen deprivation, radiation therapy to the lumbar and sacral spine, and stereotactic radiosurgery to 3 intracranial foci of disease. He died 14 months after leptomeningeal recurrence. Autopsy showed diffuse spinal leptomeningeal disease, leptomeningeal based intracranial lesions, and no other metastasis.

**Conclusions:**

The cause for solitary leptomeningeal recurrence in this patient is unknown. Although there may be many possible mechanisms, we speculate that it could be related to his initial treatment with cytotoxic gene therapy along with radiation therapy and androgen deprivation.

## Introduction

Standard of care for nonmetastatic prostate cancer depends on risk group with options including observation, radiation therapy (RT) with or without androgen deprivation therapy, and radical prostatectomy.^1^ Although therapy is generally effective for localized disease, locoregional recurrence and distant metastasis do occur and prostate cancer remains the second leading cause of cancer mortality in men.^2^ In an attempt to improve therapeutic efficacy, cytotoxic gene therapy has been investigated as a treatment for prostate cancer. This approach relies on the transfer or insertion of a gene that codes for a protein capable of activating a prodrug to produce selective cytotoxicity.^3^

Similar to how conventional immunotherapy enhances infiltration of the tumors with lymphocytes, the intraprostatic injection of ADV/HSV-*tk* followed by ganciclovir therapy and surgery showed significant influx of CD8^+^ T lymphocytes into the tumors compared with controls.^4^ However, there was no improvement in biochemical recurrence rate in these patients as measured by prostate-specific antigen (PSA). Preclinical models showed that cytotoxic gene therapy combined with radiation therapy (gene-RT) enhanced local cytotoxicity in prostate cancer, decreased metastases, and improved survival in preclinical models compared with each treatment alone.^5^^,^^6^ A nonrandomized clinical trial evaluating gene-RT showed superior 5-year PSA relapse-free survival in the high-risk prostate cancer arm (91% vs 72%) relative to the high-risk prostate cancer arm in the simultaneously conducted nonrandomized clinical trial by Zelefsky et al; both trials involved dose-escalated intensity modulated radiation therapy (IMRT) and short-course androgen deprivation.^7^, ^8^, ^9^, ^10^ A subsequent randomized controlled phase 3 trial (NCT 01436968) evaluating gene-RT is in progress.

Leptomeningeal metastasis from prostate adenocarcinoma is extremely rare. A total of 46 previous cases of leptomeningeal metastatic prostate adenocarcinoma have been reported in the literature.^11^, ^12^, ^13^, ^14^, ^15^, ^16^, ^17^, ^18^, ^19^, ^20^, ^21^ Only one reported a patient with solitary leptomeningeal recurrence (ie, no other systemic metastasis) of prostate adenocarcinoma- a very inexplicable situation.^16^ We report the second such case, and the first autopsy-proven solitary leptomeningeal recurrence of prostate adenocarcinoma in a patient who received gene-RT with androgen deprivation therapy.

## Case Report

A 63-year-old white man presented in January 2001 with prostatic adenocarcinoma on transurethral resection of the prostate (TURP). At the time of the TURP, the patient's PSA was 3.1 ng/mL. Pathology revealed Gleason 3 + 5 in 7 of 60 chips (approximately 10% of the specimen). At the time of consultation, he had abnormal digital rectal examination findings, with a firm nodule on the right base and induration extending to the right seminal vesicles. Bone scan, computed tomography of the pelvis and chest x-ray were negative for metastatic disease. The patient was therefore staged as T3b. The patient had a family history significant for prostate cancer in his father, grandfather and 2 uncles. He was otherwise healthy.

The patient enrolled in a phase I-II trial evaluating combined IMRT and in situ gene therapy.^7^, ^8^, ^9^ At the time of enrollment, the patient underwent a prostate biopsy revealing Gleason 3 + 4 adenocarcinoma in 4 out of 6 cores. He was treated on protocol with IMRT to the prostate and seminal vesicles, cytotoxic gene therapy with intraprostatic injection of ADV/HSV-*tk,* oral valacyclovir and concurrent androgen deprivation with leuprolide for 4 months. Gene therapy involved injections on days 0, 56, and 70. Each injection was followed by 14 days of valacyclovir. Androgen deprivation therapy consisted of flutamide starting on day 0 for 14 days and a 4-month injection of leuprolide on day 0. Radiation therapy started on day 58 and proceeded for 35 fractions to a total dose of 70 Gy. The patient finished treatment in May 2001. After treatment, the patient's PSA declined to 0.1. Posttreatment prostate biopsies were negative.

**Figure 1:**
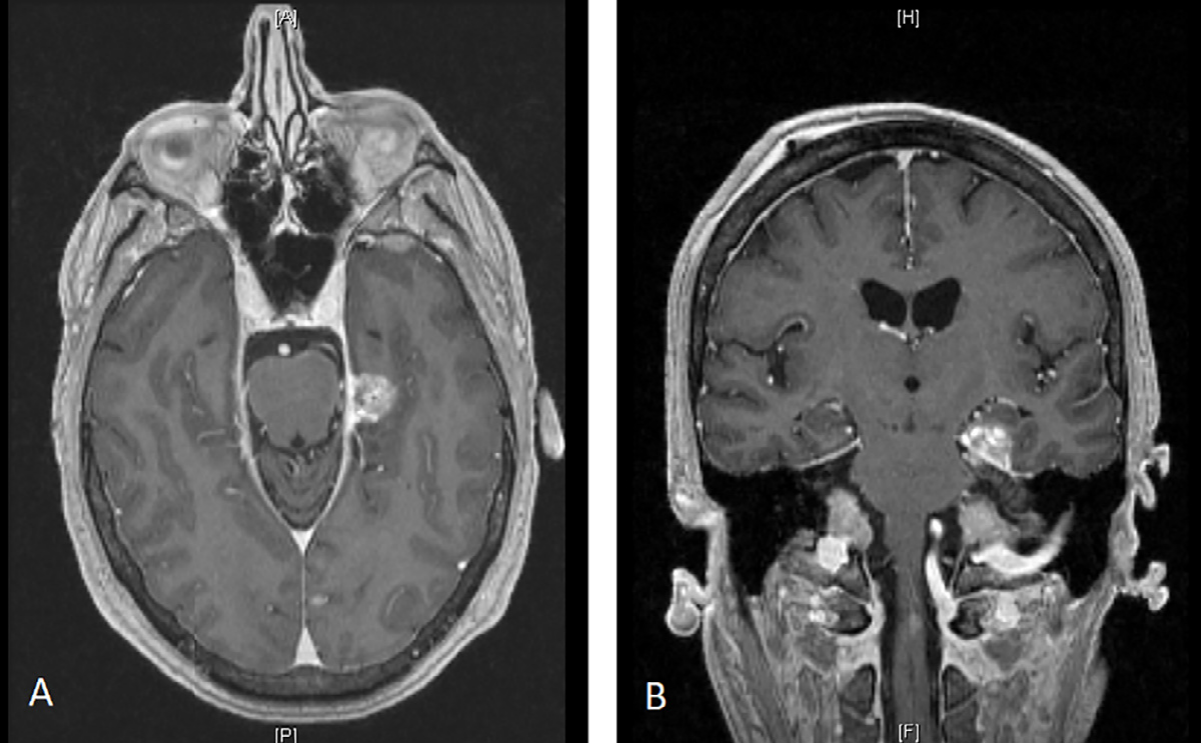
Enhancement and nodularity of a left-sided tentorial-based dural lesion. (A) Axial image. (B) Coronal image.

The patient had 37 months off all therapy with no evidence of disease until his PSA began to rise to 1 ng/mL in June 2004. Magnetic resonance imaging (MRI) of the pelvis in July 2004 was unremarkable and he was observed. In November 2004 he had back pain and tinnitus. PSA at this time was 3.1 ng/mL. MRI of the spine demonstrated a nodular appearance in the thecal sac and sacral spinal canal suggesting intradural metastatic disease. Lumbar puncture revealed cells consistent with adenocarcinoma. MRI of the brain suggested tiny foci of cranial leptomeningeal disease. The patient was treated with leuprolide, bicalutamide, and conventional radiation therapy to the lumbar and sacral spine. Chemotherapy with taxotere was deferred due to continued hormone sensitivity. The brain lesions decreased in size with androgen deprivation and were observed until September 2005 when the patient had seizures and difficulty swallowing. MRI done at that time revealed growth of a left sided tentorial-based lesion (Fig. 1), and new dural based lesions in the middle cranial fossa and right frontal parasagittal region. Spinal leptomeningeal disease was also seen diffusely with the exception of the area of prior radiation. The patient's cranial disease was treated with stereotactic radiosurgery (16 Gy to each of the 3 lesions) in November 2005. Stereotactic radiosurgery was chosen over whole brain radiation due to the absence of brain parynchymal metastasis, the desire to preserve bone marrow for systemic chemotherapy, and the ability to initiate systemic chemotherapy sooner. His neurologic symptoms resolved, and he was then treated with intrathecal cytarabine and systemic mitoxantrone.

**Figure 2:**
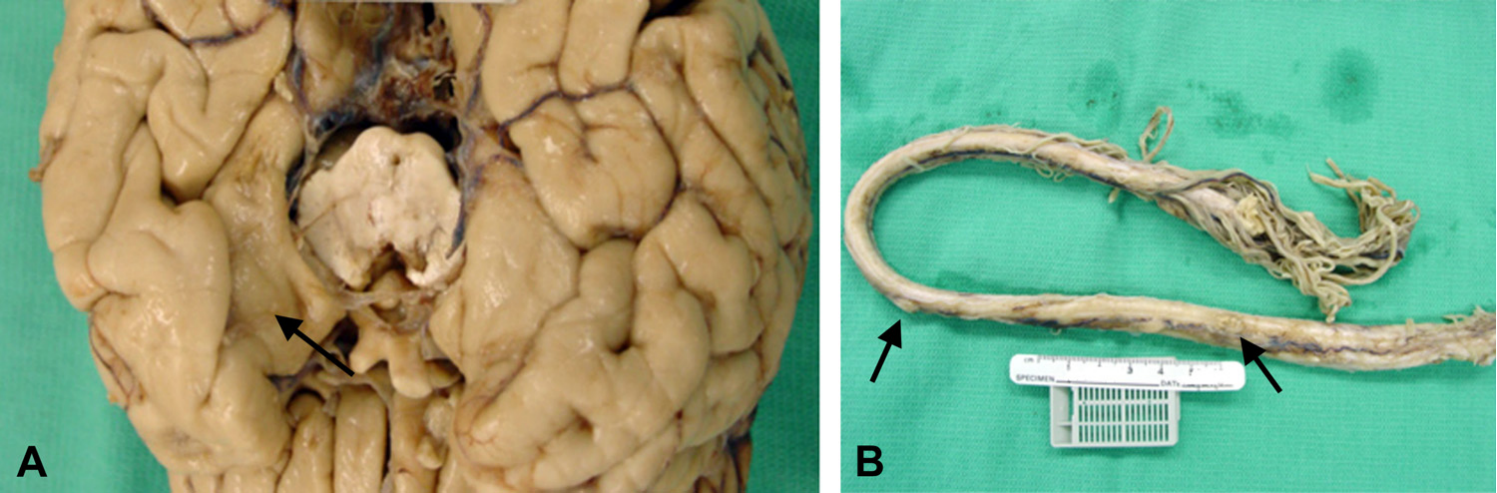
Gross photographs of brain and spinal leptomeningeal disease. (A) Brain with arrow demonstrating dural based lesion. (B) Spinal cord and cauda equina with arrow showing nodularity.

**Figure 3:**
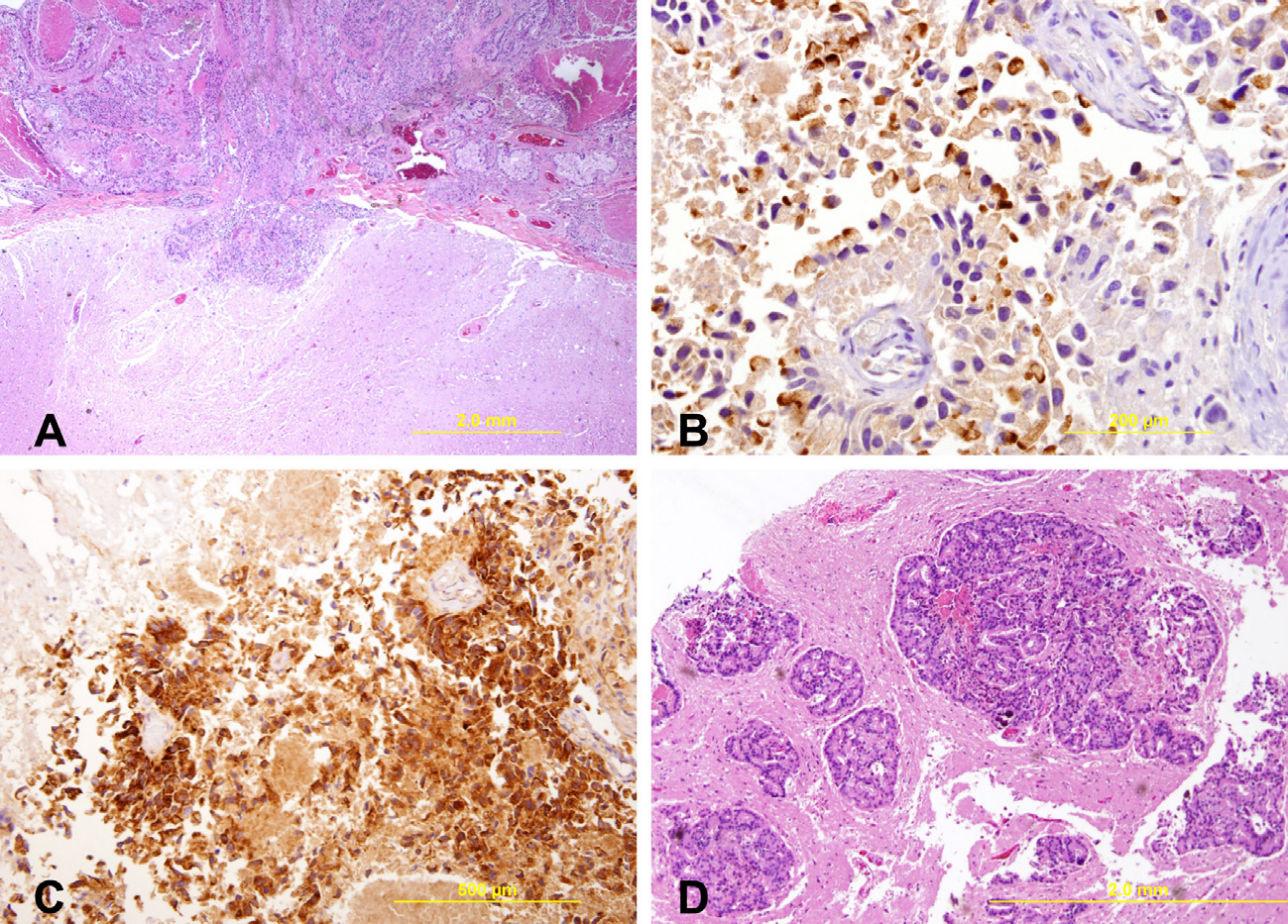
Microscopic sections. (A) Hematoxylin and eosin (H&E) staining of a nodular spinal cord lesion demonstrating prostatic adenocarcinoma. (B) Spinal cord lesion staining positive with protein-specific antigen. (C) Spinal cord lesion staining positive with [alpha]-methylacyl-CoA racemase. (D) H&E staining of the temporal lobe lesion demonstrating prostatic adenocarcinoma.

The patient was admitted to the hospital in January 2006 where he was found to have disease in the left temporal lobe, pituitary, and spinal cord. He became weaker, less responsive and died 2 weeks later. Autopsy (Fig. 2) performed the day after his death revealed 2 light tan exophytic nodules in the dura: one in the left sphenoid bone region and one near the right posterior foramen magnum. Each lesion was found to have prostatic adenocarcinoma of the ductal endometrioid type with a Gleason score 5 + 4 = 9 (Fig. 3). The remaining central nervous system was found to have an 8 mm lesion in the left temporal lobe (corresponding to the treated lesion) and a 9 mm lesion in the left parahippocampal region which was new. All brain lesions were leptomeningeal based. Diffuse leptomeningeal spinal disease was also apparent. No malignancy was found in the other organs of the body including the lymph nodes, bone, bone marrow, liver, or lungs. The testes were atrophic. Examination of the prostate revealed treatment effects without evidence of adenocarcinoma.

## Discussion

Of note, this article was written 14 years after the patient's death. The relevance of this case report was not clear at the time of his death because neither the antineoplastic role of immunotherapy nor the mechanisms of the abscopal effect were well established at that time. Due to the contemporary prominence of immunotherapy and the improved understanding of the abscopal effect, we recently determined that this case report would add value to the literature.

Leptomeningeal disease is a rare manifestation of prostate cancer with only 46 previously reported cases in the literature.^11^, ^12^, ^13^, ^14^, ^15^, ^16^, ^17^, ^18^, ^19^, ^20^, ^21^ In a large review of 41,830 prostate cancer patients at a MD Anderson Cancer Center, only 7 patients (<0.02%) had leptomeningeal disease.^18^ An Italian study evaluating 943 patients with castrate resistant prostate cancer who received docetaxel found 9 patients (0.95%) with leptomeningeal disease.^15^ This latter study had stricter inclusion criteria selecting for higher risk patients, which is largely responsible for the higher rate compared with the MD Anderson study.

There are 3 general mechanisms of leptomeningeal spread: direct extension (from brain parenchyma, dura, or bone), hematologic spread, or perineural extension.^22^ At autopsy, the patient had no parenchymal brain metastasis and no extraneuraxial metastasis. Therefore, the patient's metastases either started in the dura mater and extended inwardly into the leptomeninges or started in the leptomeninges from hematogenous spread and extended outwardly into the dura mater. From an anatomic perspective, the former situation would not require crossing the blood-brain barrier (BBB) while the latter would.

Much remains unknown about the molecular mechanisms of tumor colonization in the leptomeninges.^22^ However, a recent study did show that once in the CSF, cancer cells upregulate complement component 3, which leads to disruption of the BBB and entry of plasma growth factors into the cerebrospinal fluid, promoting cancer cell growth.^23^

There are no large autopsy case series for any malignancy defining the rate of solitary leptomeningeal disease recurrence relative to leptomeningeal disease occurring concurrent with other sites of disease. Two prior case series have shown a 10% to 20% rate of solitary leptomeningeal disease in solid tumors; however, neither study had full staging performed at the time of leptomeningeal diagnosis and neither study involved autopsy evaluation.^24^^,^^25^ Therefore, the frequency of this phenomenon may be much lower than reported.

Recurrent metastatic prostate adenocarcinoma most frequently involves lymph nodes or bones.^26^ The patient's treatment with gene-RT may have had a role in his unique pattern of relapse. Recently as the antineoplastic effects of immunotherapy have become clinically relevant and the impact of gene-RT on the immune system has become clearer, gene-RT is now colloquially referred to as gene-mediated cytotoxic immunotherapy and it has been examined in many disease sites as a form of immunotherapy.^27^ Compared with baseline levels, gene-RT has been shown to change the quantitative distribution of certain immune cells in the blood.^28^^,^^29^ These changes include decreases in NK cells and CD19^+^ B cells, increases in total CD8^+^ and CD4^+^ T cells, and increases in activated (ie, HLA-DR^+^) CD8^+^ and CD4^+^ T cells.^29^ Although the downstream consequences are poorly understood, these immunologic alterations could conceivable be responsible for this patient's pattern of recurrence.

The patient had Gleason score 3 + 5 at the time of TURP and Gleason score 3 + 4 at the time of biopsy. This discordance is likely due to sampling error. At the time of autopsy, there was no longer any adenocarcinoma in the prostate and the leptomeningeal disease was found to have a Gleason score of 5 + 4. The former suggests local response to definitive prostate cancer treatment, whereas the latter suggests increased aggressiveness of the distant disease.

The metastatic process in general is complex and incompletely understood. Once in the vasculature, extravasation and colonization are necessary for a metastasis to occur.^30^ It is possible that the immunologic alterations induced by gene-RT may have made the leptomeninges more vulnerable to tumor cell extravasation and/or colonization. Alternatively, gene-RT may have altered the prostate cancer cell line in such a way that facilitated extravasation or colonization at the leptomeninges.

Another plausible explanation is that the immunologic alterations induced by gene-RT may have been able to protect systemic organs but were unable to penetrate the BBB. Although the effect of gene-RT relative to the BBB is unknown, both cellular and antibody mediated immune entry into the central nervous system are highly regulated under normal physiology.^31^^,^^32^ The abscopal effect is a long-known phenomenon whereby local radiation therapy alone can induce clinical responses at distant sites.^33^^,^^34^ It has been suggested that the BBB may be a limitation of the abscopal effect.^35^ Presuming that the immunomodulatory effects of gene-RT are essentially an amplified version of the abscopal effect from radiation therapy alone, it stands to reason that antitumor immune modulators may have been unable to cross the BBB in this patient.

## Conclusions

We presented the second published case of solitary leptomeningeal recurrence of prostate adenocarcinoma and the first such case in a patient receiving gene-RT. The cause for solitary leptomeningeal recurrence in this patient is unknown. Although there may be many possible mechanisms, we speculate that it could be related to his initial treatment with cytotoxic gene therapy along with radiation therapy and androgen deprivation.

## References

[1] Schaeffer E, Srinivas S, Antonarakis ES (2020). NCCN guidelines prostate cancer version 2. https://www.nccn.org/professionals/physician_gls/pdf/prostate.pdf.

[2] Siegel RL, Miller KD, Jemal A. (2020). Cancer statistics, 2020. CA Cancer J Clin.

[3] Teh BS, Aguilar-Cordova E, Vlachaki MT (2002). Combining radiotherapy with gene therapy (from the bench to the bedside): A novel treatment strategy for prostate cancer. Oncologist.

[4] Ayala G, Satoh T, Li R (2006). Biological response determinants in HSV-tk + ganciclovir gene therapy for prostate cancer. Mol Ther.

[5] Tetzlaff MT, Teh BS, Timme TL (2006). Expanding the therapeutic index of radiation therapy by combining in situ gene therapy in the treatment of prostate cancer. Technol Cancer Res Treat.

[6] Tetzlaff MT, Teh BS, Timme TL (2006). Expanding the therapeutic index of radiation therapy by combining in situ gene therapy in the treatment of prostate cancer. Technol Cancer Res Treat.

[7] Teh BS, Aguilar-Cordova E, Kernen K (2001). Phase I/II trial evaluating combined radiotherapy and in situ gene therapy with or without hormonal therapy in the treatment of prostate cancer- a preliminary report. Int J Radiat Oncol Biol Phys.

[8] Teh BS, Ayala G, Aguilar L (2004). Phase I-II trial evaluating combined intensity-modulated radiotherapy and in situ gene therapy with or without hormonal therapy in treatment of prostate cancer- interim report on PSA response and biopsy data. Int J Radiat Oncol Biol Phys.

[9] Teh BS, Ishiyama H, Mai W (2015). Long-term outcome of a phase II trial using immunomodulatory in situ gene therapy in combination with intensity-modulated radiotherapy with or without hormonal therapy in the treatment of prostate cancer. J Radiat Oncol.

[10] Zelefsky MJ, Chan H, Hunt M (2006). Long-term outcome of high dose intensity modulated radiation therapy for patients with clinically localized prostate cancer. J Urol.

[11] Matsui H, Terahata N, Kanamori M. (1995). Diffuse spinal leptomeningeal metastases from prostatic cancer. A case report. Int Orthop.

[12] Chamberlain MC, Kormanik PA, Barba D. (1997). Complications associated with intraventricular chemotherapy in patients with leptomeningeal metastases. J Neurosurg.

[13] Bernstein WB, Kemp JD, Kim GS (2008). Diagnosing leptomeningeal carcinomatosis with negative CSF cytology in advanced prostate cancer. J Clin Oncol.

[14] Orphanos G, Ioannidis G, Michael M (2009). Prostate-specific antigen in the cerebrospinal fluid: a marker of local disease. Med Oncol.

[15] Caffo O, Gernone A, Ortega C (2012). Central nervous system metastases from castration-resistant prostate cancer in the docetaxel era. J Neurooncol.

[16] Zhang M, Mahta A, Kim RY (2012). Durable remission of leptomeningeal metastases from hormone-responsive prostate cancer. Med Oncol.

[17] Cante D, Franco P, Sciacero P (2013). Leptomeningeal metastasis from prostate cancer. Tumori.

[18] Yust-Katz S, Mathis S, Groves MD. (2013). Leptomeningeal metastases from genitourinary cancer: The University of Texas MD Anderson Cancer Center experience. Med Oncol.

[19] Ng JY, Ng JY. (2018). Leptomeningeal metastases in hormone refractory prostate cancer. Cureus.

[20] Carroll RD, Leigh EC, Curtis Z (2019). A case of leptomeningeal carcinomatosis from aggressive metastatic prostate cancer. Case Rep Oncol.

[21] Neeman E, Salamon N, Rettig M. (2020). Leptomeningeal carcinomatosis of prostate cancer: A case report and review of the literature. Rev Urol.

[22] Wang N, Bertalan MS, Brastianos PK. (2018). Leptomeningeal metastasis from systemic cancer: Review and update on management. Cancer.

[23] Boire A, Zou Y, Shieh J (2017). Complement component 3 adapts the cerebrospinal fluid for leptomeningeal metastasis. Cell.

[24] Clarke JL, Perez HR, Jacks LM (2010). Leptomeningeal metastases in the MRI era. Neurology.

[25] Kaplan JG, DeSouza TG, Farkash A (1990). Leptomeningeal metastases: Comparison of clinical features and laboratory data of solid tumors, lymphomas and leukemias. J Neurooncol.

[26] Giesel FL, Knorr K, Spohn F (2019). Detection efficacy of ^18^F-PSMA-1007 PET/CT in 251 patients with biochemical recurrence of prostate cancer after radical prostatectomy. J Nucl Med.

[27] Wheeler LA, Manzanera AG, Bell SD (2016). Phase II multicenter study of gene-mediated cytotoxic immunotherapy as adjuvant to surgical resection for newly diagnosed malignant glioma. Neuro Oncol.

[28] Satoh T, Teh BS, Timme TL (2004). Enhanced systemic T-cell activation after in situ gene therapy with radiotherapy in prostate cancer patients. Int J Radiat Oncol Biol Phys.

[29] Fujita T, Teh BS, Timme TL (2006). Sustained long-term immune responses after in situ gene therapy combined with radiotherapy and hormonal therapy in prostate cancer patients. Int J Radiat Oncol Biol Phys.

[30] Lambert AW, Pattabiraman DR, Weinberg RA. (2017). Emerging biological principles of metastasis. Cell.

[31] Pachter JS, de Vries HE, Fabry Z. (2003). The blood-brain barrier and its role in immune privilege in the central nervous system. J Neuropathol Exp Neurol.

[32] Deeken JF, Löscher W. (2007). The blood-brain barrier and cancer: Transporters, treatment, and Trojan horses. Clin Cancer Res.

[33] Chen AC, Butler EB, Lo SS (2015). Radiotherapy and the abscopal effect: Insight from the past, present, and future. J Radiat Oncol.

[34] Formenti SC, Demaria S. (2009). Systemic effects of local radiotherapy. Lancet Oncol.

[35] Ishiyama H, Teh BS, Ren H (2012). Spontaneous regression of thoracic metastases while progression of brain metastases after stereotactic radiosurgery and stereotactic body radiotherapy for metastatic renal cell carcinoma: Abscopal effect prevented by the blood-brain barrier?. Clin Genitourin Cancer.

